# Oxygen carriers affect kidney immunogenicity during *ex-vivo* machine perfusion

**DOI:** 10.3389/frtra.2023.1183908

**Published:** 2023-06-16

**Authors:** Tamina Rother, Carina Horgby, Katharina Schmalkuche, Jonathan M. Burgmann, Fabian Nocke, Johannes Jägers, Jessica Schmitz, Jan Hinrich Bräsen, Miriam Cantore, Franck Zal, Katja B. Ferenz, Rainer Blasczyk, Constanca Figueiredo

**Affiliations:** ^1^ Institute of Transfusion Medicine and Transplant Engineering, Hannover Medical School, Hannover, Germany; ^2^ Institute of Physiology, University Hospital Essen, University of Duisburg-Essen, Essen, Germany; ^3^ Department of Biomedicine, Aarhus University, Aarhus, Denmark; ^4^ Nephropathology Unit, Institute of Pathology, Hannover Medical School, Hannover, Germany; ^5^ Hemarina SA, Aéropôle Centre, Morlaix, France; ^6^ CeNIDE (Center for Nanointegration Duisburg-Essen), University of Duisburg-Essen, Duisburg, Germany

**Keywords:** oxygen carriers, normothermic *ex-vivo* machine perfusion, kidney preservation, organ immunogenicity, hemarina-M101, HEMO_2_Life, perfluorocarbon-based oxygen carriers, transplantation

## Abstract

Normothermic *ex-vivo* machine perfusion provides a powerful tool to improve donor kidney preservation and a route for the delivery of pharmacological or gene therapeutic interventions prior to transplantation. However, perfusion at normothermic temperatures requires adequate tissue oxygenation to meet the physiological metabolic demand. For this purpose, the addition of appropriate oxygen carriers (OCs) to the perfusion solution is essential to ensure a sufficient oxygen supply and reduce the risk for tissue injury due to hypoxia. It is crucial that the selected OCs preserve the integrity and low immunogenicity of the graft. In this study, the effect of two OCs on the organ's integrity and immunogenicity was evaluated. Porcine kidneys were perfused *ex-vivo* for four hours using perfusion solutions supplemented with red blood cells (RBCs) as conventional OC, perfluorocarbon (PFC)-based OC, or Hemarina-M101 (M101), a lugworm hemoglobin-based OC named HEMO_2_life®, recently approved in Europe (i.e., CE obtained in October 2022). Perfusions with all OCs led to decreased lactate levels. Additionally, none of the OCs negatively affected renal morphology as determined by histological analyses. Remarkably, all OCs improved the perfusion solution by reducing the expression of pro-inflammatory mediators (IL-6, IL-8, TNFα) and adhesion molecules (ICAM-1) on both transcript and protein level, suggesting a beneficial effect of the OCs in maintaining the low immunogenicity of the graft. Thus, PFC-based OCs and M101 may constitute a promising alternative to RBCs during normothermic *ex-vivo* kidney perfusion.

## 1. Introduction

Transplantation is the preferable treatment for patients suffering of end-stage renal diseases (1). Despite the success of kidney transplantation (KTx), ischemia-reperfusion injury remains a relevant hurdle and determines the KTx outcome. In fact, improving donor kidney preservation is important for organ reconditioning and long-term survival (2). *Ex-vivo* kidney machine perfusion (EVKP) strategies at normothermic (3) and subnormothermic (4) conditions have contributed to a significant reduction of warm and cold ischemic time and mitigation of ischemia-reperfusion injury (5, 6). Moreover, EVKP offers the opportunity for the development of new organ interventions by combination with therapies to directly manipulate and improve organ quality or function (7). However, organs perfused at normothermic temperatures have a high metabolic demand requiring an adequate tissue oxygenation to avoid hypoxia (8). The use of cellular or acellular perfusion solutions supplemented with oxygen carriers (OCs) may provide sufficient oxygen supply and prevent hypoxic tissue injury or increase of immunogenicity prior KTx (9, 10).

Red blood cell (RBC) concentrates have been traditionally used as cellular perfusion solution with hemoglobin as oxygen-carrying component (11). Nevertheless, RBC concentrates have limitations in clinical practice, including hemolysis during prolonged perfusions, deterioration of the oxygen-carrying capacity of RBCs, and activation of pro-inflammatory immune cells (12). Hence, alternative OCs have been developed as urgently awaited RBC replacement approach. In addition, those OCs usually exhibit beneficial features such as higher oxygen- and carbon dioxide-carrying capacity and a lower antigenicity (13, 14).

Since the late 20th century, two groups of extensively evaluated OCs became a promising alternative to RBC concentrates not only in the context of human transfusions, but also for normothermic and subnormothermic *ex-vivo* organ perfusion: Hemarina-M101 (M101) and perfluorocarbons (PFC). M101 is a natural extracellular biopolymer hemoglobin derived from the marine invertebrate *Arenicola marina*, which demonstrates a powerful oxygenating ability and safety in several preclinical studies and during clinical trials (15, 16). Its capability of being low immunogenic, nontoxic, and its intrinsic superoxide dismutase-like activity with antioxidant effects suggest a remarkable potential towards improving organ preservation (15, 17). M101 is characterized by their ability to offload oxygen more efficiently in the plasma then RBCs since they do not interfere with the cell membrane, resulting in an improved tissue oxygenation (18). The first transplantation-related report on M101 appeared in 2011 by Thuillier et al., showing its beneficial effects during static cold storage (SCS) by decreasing chronic fibrosis and organ dysfunction after KTx (17). Other preclinical trials on porcine kidneys indicated an improved short- and long-term functional outcome, an ameliorated graft recovery, and a maintained tissue integrity after hypothermic machine perfusion using M101 (19, 20). Promising results were also achieved in preclinical liver transplantation studies, where M101 was used as an additive to the SCS preservation solution. M101 was associated with higher ATP levels, indicating improved mitochondrial function, reduced oxidative stress levels, lower pro-inflammatory immune responses, and attenuated ischemia-reperfusion injuries (21). In addition, a recent study showed that M101 indirectly alleviates the complement activation by reducing ischemia-reperfusion injuries (14).

PFCs are hydrocarbons whose hydrogen groups were replaced by fluorine atoms, demonstrating a unique capacity for dissolving respiratory gases (22). However, the hydrophobic PFC would form large aggregates within the bloodstream, which tend to create embolisms. Consequently, pure PFCs are not suitable for intravenous injection and require emulsification or encapsulation e.g., using a membrane consisting of human serum albumin (23). PFCs and M101 are substantially smaller than RBCs, allowing them to enter and pass partially obstructed vessels, thereby maintaining oxygen supply to the peripheral tissue (22, 24). Additionally, oxygen loading and unloading of PFCs occur twice as fast as in RBCs and the oxygen extraction rate is 3-fold higher (22). In the last 30 years, Brasile et al. published several studies showing promising findings during *ex-vivo* machine perfusion with PFC emulsions using canine, porcine, and discarded human kidneys (25). Recently, PFC-based OCs were supplemented during porcine *ex-vivo* lung perfusion, which demonstrated better preservation of mitochondrial function and improved gas transport following transplantation (26). PFC also attenuated ischemia-reperfusion injury and prolonged postoperative survival in liver transplanted grafts (27).

Different studies have reported an inflammatory cytokine storm caused by *ex-vivo* organ perfusions, which is likely to be responsible for immune cell activation and mobilization after organ transplantation (28, 29). This study investigated the beneficial effects of PFC-based OCs and M101 in preventing the upregulation of parameters associated with organ immunogenicity during normothermic EVKP.

## 2. Materials and methods

### 2.1. Experimental animals and kidney retrieval

Animal experiments were approved by the supervisory authority (LAVES-Niedersächsisches Landesamt für Verbraucherschutz und Lebensmittelsicherheit) according to the recommendation of their Ethics Committee and performed in compliance with the ARRIVE guidelines, the German animal welfare law, the German guidelines for animal welfare and the EU Directive 2010/63/EU.

Pigs were routinely prepared for surgery, anesthetized with Propofol i.v. and euthanized with injection of Pentobarbital i.v. (overdosed). Kidneys were retrieved rapidly after circulatory death of the pig, flushed with 200 ml cold (4°C) Custodiol (Dr. Franz Köhler Chemie GmbH, Bensheim, Germany) and laid on ice. Next, kidneys were connected to a Kidney Assist® perfusion device (XVIVO B.V., Groningen, Netherlands) via artery cannula.

### 2.2. Experimental design and groups

#### 2.2.1. Perfusion system

Kidneys were gradually rewarmed to normothermia (37°C) and perfused for four hours. The perfusion solution was oxygenated by the oxygenator connected to the carbogen reservoir (95% O_2_:5% CO_2_) with an arterial oxygen partial pressure above 500 mmHg, allowing for sufficient oxygenation of renal tissue indicated by an undepleted oxygen outflow (30, 31). Flow, vascular resistance (VR), and temperature were continuously monitored. Pressure was flow-optimized, aiming for target values of 100–150 ml/min.

#### 2.2.2. Experimental groups and perfusion solution

Four experimental groups were defined (*n *= 3 per group) with perfusion solutions differing in the addition of OCs. As perfusion medium, 500 ml STEEN Solution™ (XVivo Perfusion AB, Göteborg, Sweden) and 500 ml Ringer's solution (Deltamedica, Reutlingen, Germany) supplemented with 16.5 ml 8.4% sodium bicarbonate (B. Braun, Melsungen, Germany) and 10 ml Cefalozin (MIP Pharma, Blieskastel, Germany) were used. Kidneys of the first group were only perfused with the STEEN/Ringer-based solution and served as control. In the second kidney perfusion group, the perfusion circuit was primed with 500 ml of the STEEN/Ringer-based solution. Afterwards, the volume was filled up with 500 ml of fresh leucocyte-depleted autologous blood, which was not stored and used shortly after drawing [(Hb) = 45.75 ± 1.75 g/L]. The kidneys of the third and fourth group were perfused with either a PFC-based OC (4 vol.%) or M101 [(Hb) = 0.99 ± 0.03 g/L]. PFC-based OCs were synthesized as described previously (23) with the following adaptations: For this study, human serum albumin was used as shell material (5% Albiomin®; Biotest Pharma GmbH, Dreieich, Germany), while the core material (oxygen-carrying agent) consists of perfluorodecalin. Furthermore, subsequent to synthesis, the emulsion was centrifuged for 30 min at 2,000×g and the pellet was resuspended in the same volume of STEEN/Ringer-based solution. Table 1 summarizes the main characteristics of the different OCs used during EVKP, resulting in different properties per carrier.

**Table 1:** Physicochemical characteristics of RBCs, M101, and PFC used during EVKP.

	RBC (32–34)	M101 (35, 36)	PFC (23, 37, 38)
Composition	natural Hb (porcine); cell membrane	natural Hb (*Arenicola marina*); biopolymer	Albumin-derived perfluorodecalin-filled nanocapsules
Size	7,000 nm	15 × 25 nm	500–1,000 nm (in STEEN Solution)
Temperature range (for EVKP)	21–37°C	4–37°C	4–37°C
pH range	6.3–7.8	5.5–7.8	7.0–7.5
Oxygen-binding sites	4	156	no covalent binding
Pharmacokinetics	MW = 64 kDa T ½ = ∼ 28 days p50 = 28.90 ± 1.54 mmHg	MW = 3,600 kDa T ½ = 50 h p50 = 7.05 ± 0.93 mmHg	T ½ = ∼ 6 h no saturation of O_2_
Concentration of OC in the experiments	[Hb] = 45.75 ± 1.75 g/L	[Hb] = 0.99 ± 0.03 g/L	4 vol.% PFC
Colloid osmotic pressure	25 mmHg*	25 mmHg*	25 mmHg*
Oxygen-carrying capacity (at pO_2_ 500 mmHg)	62.22 ml O_2_/L solution	52.24 ml O_2_/L solution	11.00 ml O_2_/L emulsion

Hb, hemoglobin; MW, molecular weight; [Hb], concentration of hemoglobin or its equivalent; T ½, half-life *in-vivo*; p50, partial pressure of oxygen necessary to saturate half of the oxygen-binding sites.

* Colloid osmotic pressure is regulated by STEEN Solution containing albumin, which is used as the basis perfusion medium in all experimental groups and simulates physiologic conditions for the kidney.

### 2.3. Perfusate analyses

#### 2.3.1. Lactate levels

Perfusate samples were taken at different time points (0, 60, 120, 180, and 240 min) after perfusion start, centrifuged at 1,500 rpm for 10 min and stored at −80°C until analyses. Lactate levels in the perfusion solution were evaluated by using the Lactate-Glo Assay System (Promega, Madison, Wisconsin, USA) according to the manufacturer's protocol. Briefly, perfusate samples were diluted 1:50 with Dulbecco's Phosphate Buffered Saline (Lonza, Walkersville, Maryland, USA). Relative luminescence units (RLU) were measured with Lumat LB 9,507 luminometer (Berthold Technologies, Zug, Switzerland) and lactate concentrations were calculated by extrapolation using a standard curve. The basal lactate concentration was subtracted from the values detected at the different time points for normalization.

#### 2.3.2. Aspartate aminotransferase levels

Aspartate aminotransferase (AST) activity was measured in perfusate samples of the different kidney perfusion groups using the Aspartate Aminotransferase Activity Assay Kit (Sigma-Aldrich, St. Louis, Missouri, USA). Perfusate samples were diluted 1:6 with AST Assay Buffer. According to manufacturer's instructions, generation of the colorimetric reaction product was determined at an absorbance of 450 nm by the Synergy 2 Multi-detection microplate reader (BioTek Instruments, Inc., Winooski, Vermont, USA). AST activity levels detected at the different time points were normalized to the basal level.

#### 2.3.3. Cytokine secretion profile

Cytokine profile was determined using Luminex technology (Luminex Corp., Austin, Texas, USA), quantifying the expression of porcine interleukin (IL)-1β, tumor necrosis factor (TNF)α, IL-6, and IL-8 in perfusate samples by Luminex® 100/200 analyzer (Luminex Corp.). Samples and standards were prepared according to the manufacturer's instructions. Cytokine concentrations were calculated using the Xponent software version 3.1 (Luminex Corp.).

#### 2.3.4. Hemolysis levels

Hemolysis level of RBCs was measured in perfusate samples of kidneys exposed to blood (group 2) by detecting free hemoglobin. Perfusate samples of the STEEN/Ringer-based solution served as negative control. Absorbance at 405 nm was determined with the Synergy 2 Multi-detection microplate reader (BioTek Instruments, Inc.) and optical density (OD) units were corrected by background subtraction.

### 2.4. Real-time polymerase chain reaction

Tissue samples of perfused and non-perfused kidneys stored in RNAlater™ Stabilization Solution (Merck, Darmstadt, Germany) were used for total RNA isolation (RNease Mini Kit; Qiagen, Hilden, Germany) and reverse transcribed to cDNA using a High-Capacity cDNA Reverse Transcription Kit (Applied Biosystems, Waltham, Massachusetts, USA). As markers for endothelial activation, transcript levels of intercellular adhesion molecule 1 (ICAM-1; Ss03392384_m1), vascular cell adhesion molecule 1 (VCAM-1; Ss03390909_m1), heat shock protein 70 (Hsp70; Ss03387784_u1), IL-6 (Ss03384604_u1), IL-8 (Ss03392437), TNFα (Ss03391318_g1), β2-microglobulin (β2 m; Ss03391154_m1), and swine leukocyte antigen-DRα (SLA-DRα; Ss03389942_m1; all from Thermo Fisher Scientific, Waltham, Massachusetts, USA) were examined by quantitative polymerase chain reaction (qPCR). GAPDH (Ss03375629_u1) was used as endogenous control for normalization of mRNA levels.

### 2.5. Histological evaluation

After four hours of perfusion, kidney samples consisting of cortex and medulla regions were collected, fixed in 4% paraformaldehyde solution, and embedded in paraffin. Sections of three µm thickness were stained with hematoxylin and eosin and renal structure was visualized with Keyence microscope (Keyence, Itasca, Illinois, USA) and evaluated by a pathologist.

### 2.6. Statistical analyses

Statistical analyses were performed using GraphPad Prism v5.0 (GraphPad Software, Inc., San Diego, California, USA). Data are presented as the mean ± standard deviation and one-way ANOVA with multiple comparisons was applied to compare relative quantification (RQ) values between the groups. A two-way ANOVA was used for comparisons of data with two categorical variables (e.g., perfusion time, applied OC). *p*-Values of <0.05 were considered statistically significant and defined as *p < 0.05, **p < 0.01, ***p < 0.001 and ****p < 0.0001.

## 3. Results

### 3.1. Effect of OCs on EVKP parameters

**Figure 1:**
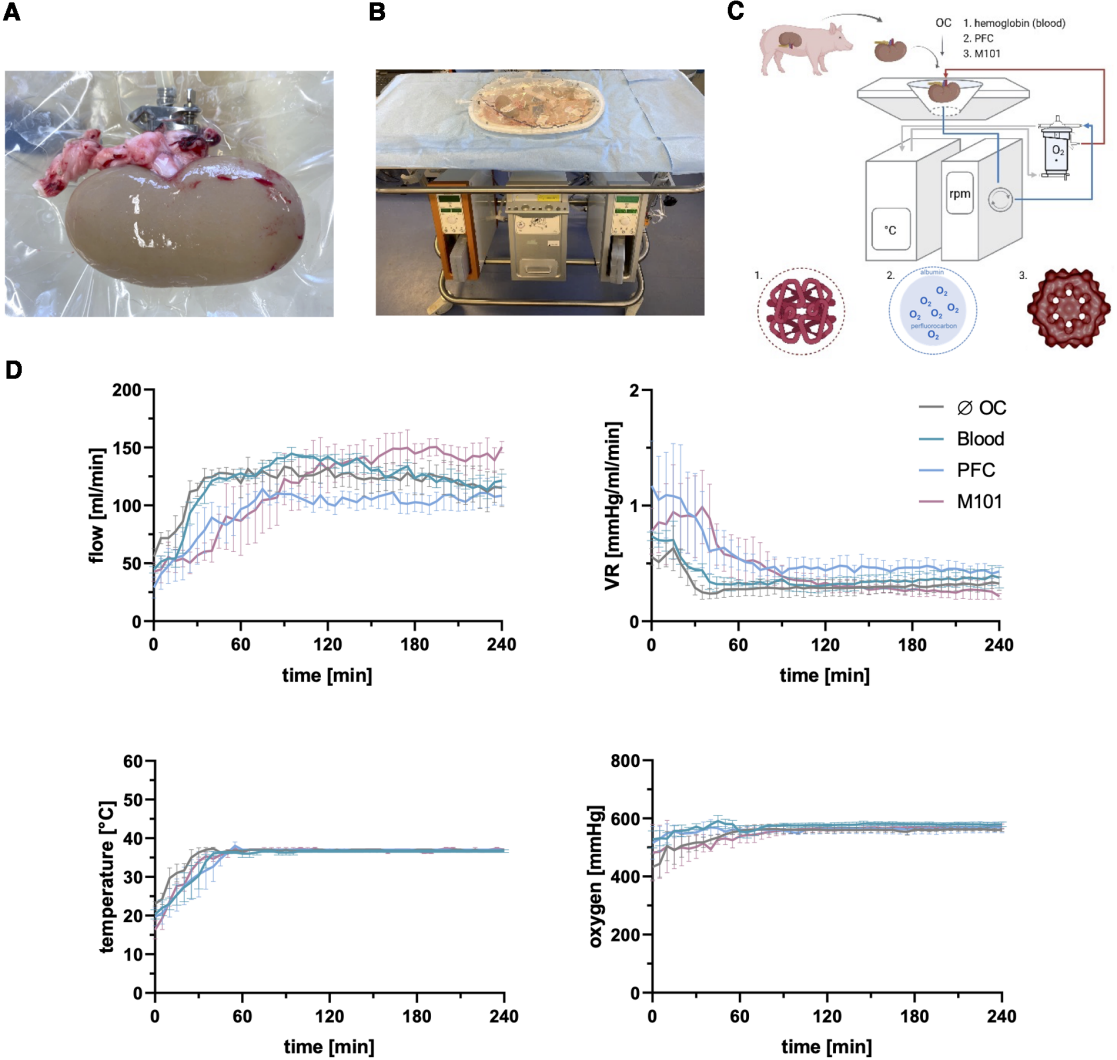
Normothermic *ex-vivo* kidney machine perfusion (EVKP) with perfluorocarbon (PFC)-based oxygen carriers (OCs) and M101. (**A**) Kidney connected to the Kidney Assist® perfusion system via the renal artery. (**B**) Kidney Assist® perfusion system. (**C**) Schematic representation of the EVKP system with its main components: perfusion reservoir, thermo unit, pump unit, and oxygenator. The oxygenated and deoxygenated perfusion solution circulates as red and blue line through the system, respectively. The following OCs are supplemented to the STEEN/Ringer-based solution: blood, PFC-based OC (PFC), and Hemarina-M101 (M101) (created with BioRender.com). (**D**) Graphs display flow rate, vascular resistance (VR), temperature, and oxygen partial pressure of kidneys perfused with different OCs over time. Graphs are represented as mean and standard deviation (*n *= 3).

The use of OCs during *ex-vivo* organ perfusion may enable a sustained and efficient graft oxygenation during *ex-vivo* preservation. We perfused porcine kidneys (Figures 1A,B) *ex-vivo* with distinct groups of state-of-the-art OCs (Figure 1C) to evaluate their potential beneficial effects in terms of preserving tissue low immunogenicity and integrity. Kidneys perfused without OCs or blood reached average flow rates of 118.36 ± 16.56 ml/min and 119.93 ± 24.44 ml/min, respectively, with corresponding VRs of 0.32 ± 0.08 mmHg/ml/min and 0.38 ± 0.10 mmHg/ml/min. The addition of alternative OCs led to comparable flow rates of 95.81 ± 20.02 ml/min with PFC-based OCs and 115.27 ± 35.16 ml/min with M101 and VR levels of 0.56 ± 0.21 mmHg/ml/min and 0.45 ± 0.24 mmHg/ml/min, respectively. Even though the average flow rates and VR levels did not significantly differ between the groups, slight differences (*p *< 0.05–0.01) could be detected within the first 45 min of perfusion between individual groups. However, after the initial stabilization period, comparable perfusion parameters were observed regardless of the OC used. After warming up the system, all groups of *ex-vivo* perfused kidneys reached normothermic temperatures of 36–37°C, which remained constant during the entire perfusion time. An arterial oxygen partial pressure above 500 mmHg was achieved after 30 min of perfusion in all groups and also remained constant over time. No significant differences were found between the average oxygen partial pressures of different groups (without OC: 547.41 ± 28.94 mmHg; blood: 572.73 ± 13.56 mmHg; PFC-based OC: 560.21 ± 9.68 mmHg; M101: 549.55 ± 27.89 mmHg) (Figure 1D). However, the use of a pump system resulted in significantly increased RBC hemolysis levels in the perfusates of kidneys exposed to blood, indicated by an OD of 1.01 ± 0.10 after 240 min of EVKP compared to an initial value of 0.31 ± 0.20 (Supplementary Figure S1).

### 3.2. Quality assessment of the kidney during EVKP

**Figure 2:**
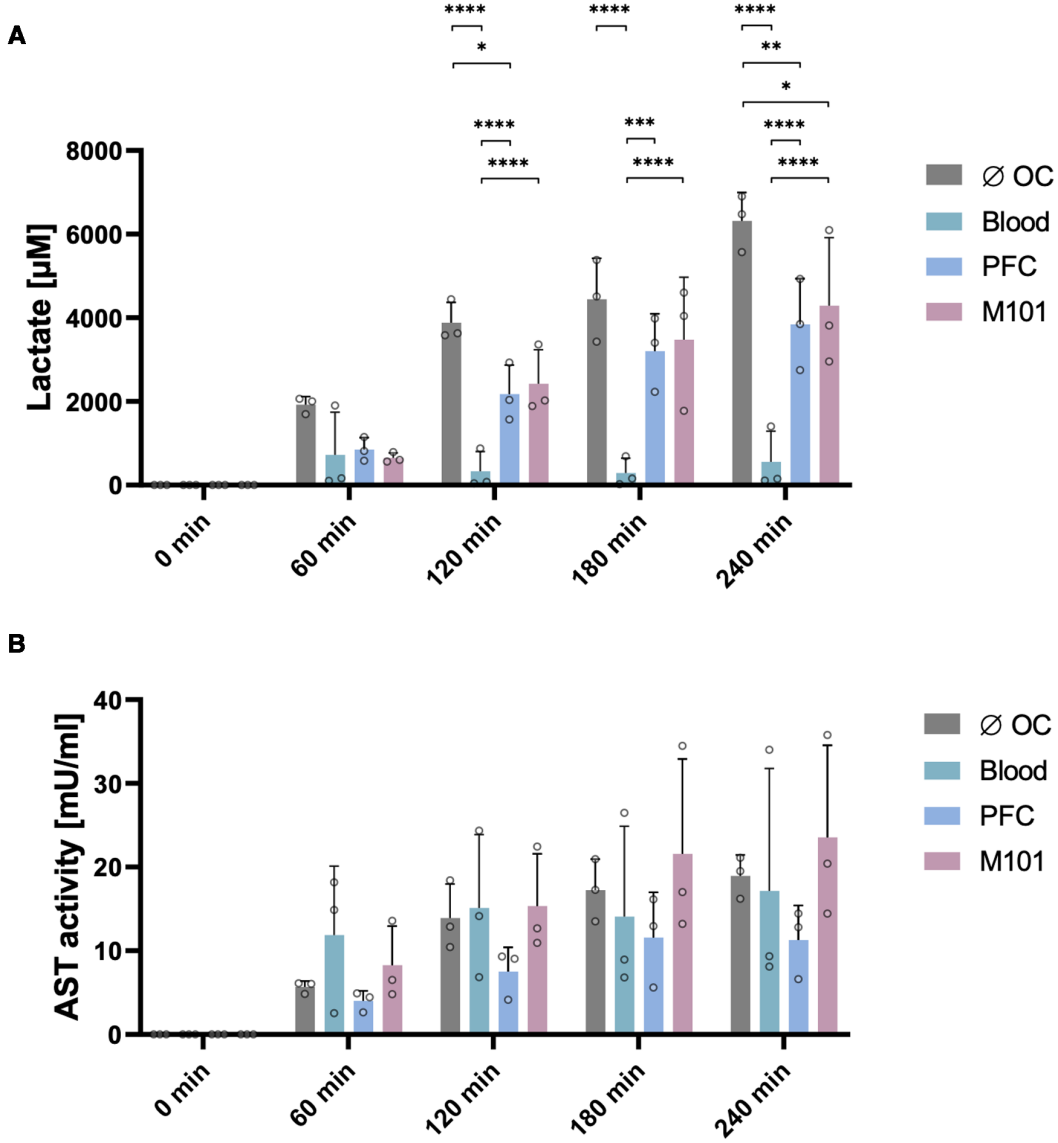
Quality assessment of the kidney during normothermic EVKP using OCs. Lactate levels (**A**) and aspartate aminotransferase (AST) activity (**B**) were quantified in perfusates of control kidneys and kidneys exposed to OCs at different time points (0, 60, 120, 180, and 240 min). Graphs represent means and standard deviations (*n *= 3). Lactate levels differed significantly between the individual groups (*p < 0.05; **p < 0.01; ***p < 0.001; ****p < 0.0001; two-way ANOVA).

High lactate levels indicate for an insufficient supply of oxygen to renal cells preventing oxidative phosphorylation and resulting in an anaerobic metabolism. Levels of lactate in the perfusate were previously used as marker for tissue hypoxia (2) caused by cold ischemia as well as reperfusion. Lactate levels in kidneys perfused with blood did not change considerably during perfusion (T60: 722.77 ± 1,021.70 µM and T240: 554.73 ± 733.25 µM) and remained low over time. In comparison to the lactate levels measured in perfusate of kidneys perfused without OCs (6,317.99 ± 681.29 µM), the supplementation of the perfusion solution with PFC-based OCs or M101 resulted in a significantly reduced lactate accumulation after four hours of EVKP. Remarkably, lactate concentrations of 3,843.36 ± 1,090.65 µM (*p *< 0.01) and 4,290.27 ± 1,625.26 µM (*p *< 0.05) were measured in the perfusate of kidneys perfused with PFC-based OCs and M101, respectively (Figure 2A). The results suggest for less tissue injury induced by hypoxia during EVKP in the presence of OCs.

### 3.3. Assessment of renal tissue integrity after EVKP with OCs

AST is present in the cytoplasm and mitochondria of cells. During tissue injury, AST is released mainly from the cytoplasm, whereby the amount of AST directly correlates with the extent of tissue damage (39). AST activity levels increased in all four groups during EVKP. No significant differences were observed between the different groups (without OC: 18.95 ± 2.49 mU/ml; blood: 17.16 ± 14.61 mU/ml; PFC-based OC: 11.29 ± 4.13 mU/ml; M101: 23.56 ± 11.01 mU/ml) after four hours of EVKP (Figure 2B).

**Figure 3:**
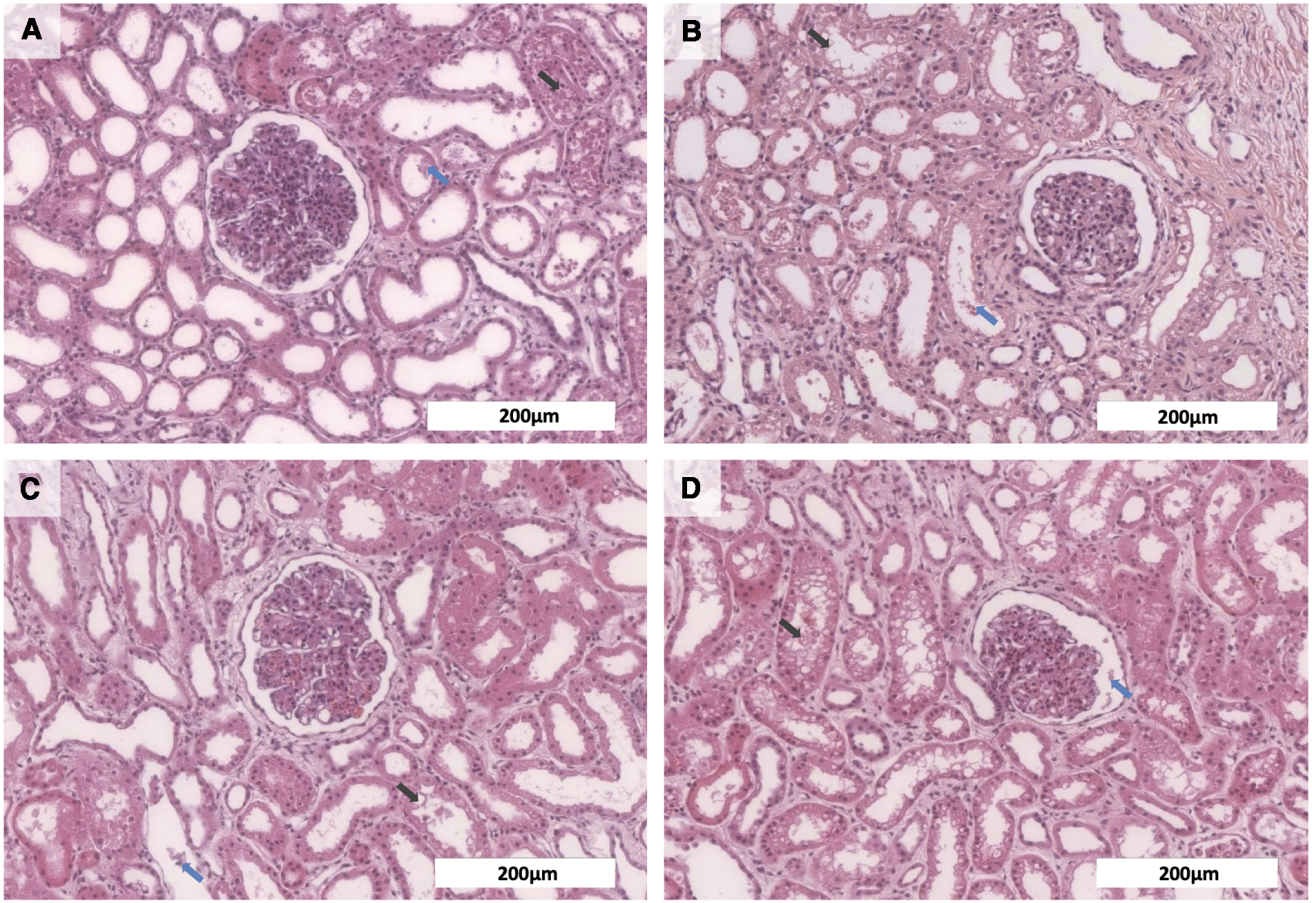
Histological analyses of kidneys after normothermic EVKP using OCs. Representative images of hematoxylin and eosin stained renal cortex regions: Kidneys perfused with no OCs (**A**), blood (**B**), PFC-based OCs (**C**), or M101 (**D**) showed necrotic cell detritus within the tubular lumen (blue arrow) as well as a mild tubular vacuolization (black arrow), indicating a potentially reversible mild to moderate acute tubular injury and minor signs of tubular necrosis. However, overall renal morphology of the individual groups was intact (scale bar: 200 µm).

Further, histopathological examinations were performed to evaluate tissue integrity after EVKP. All control kidney tissues and those perfused with OCs showed comparable findings indicating low interstitial edema as well as dilated Bowman's space and renal tubules with necrotic cell detritus within the tubular lumen. Moreover, a mild tubular vacuolization was detected in the tissues of all experimental groups. Thus, histological findings revealed a potentially reversible mild to moderate acute tubular injury and minor signs of tubular necrosis, even though renal morphology remained intact in all analyzed samples. Remarkably, the histological analyses demonstrated that the supplementation of the perfusion solution with blood, PFC-based OCs or M101 did not increase the level of tissue injury in comparison to the control group (Figure 3). These results suggest that all OC groups preserve the renal structure integrity.

### 3.4. Cytokine secretion profile

**Figure 4:**
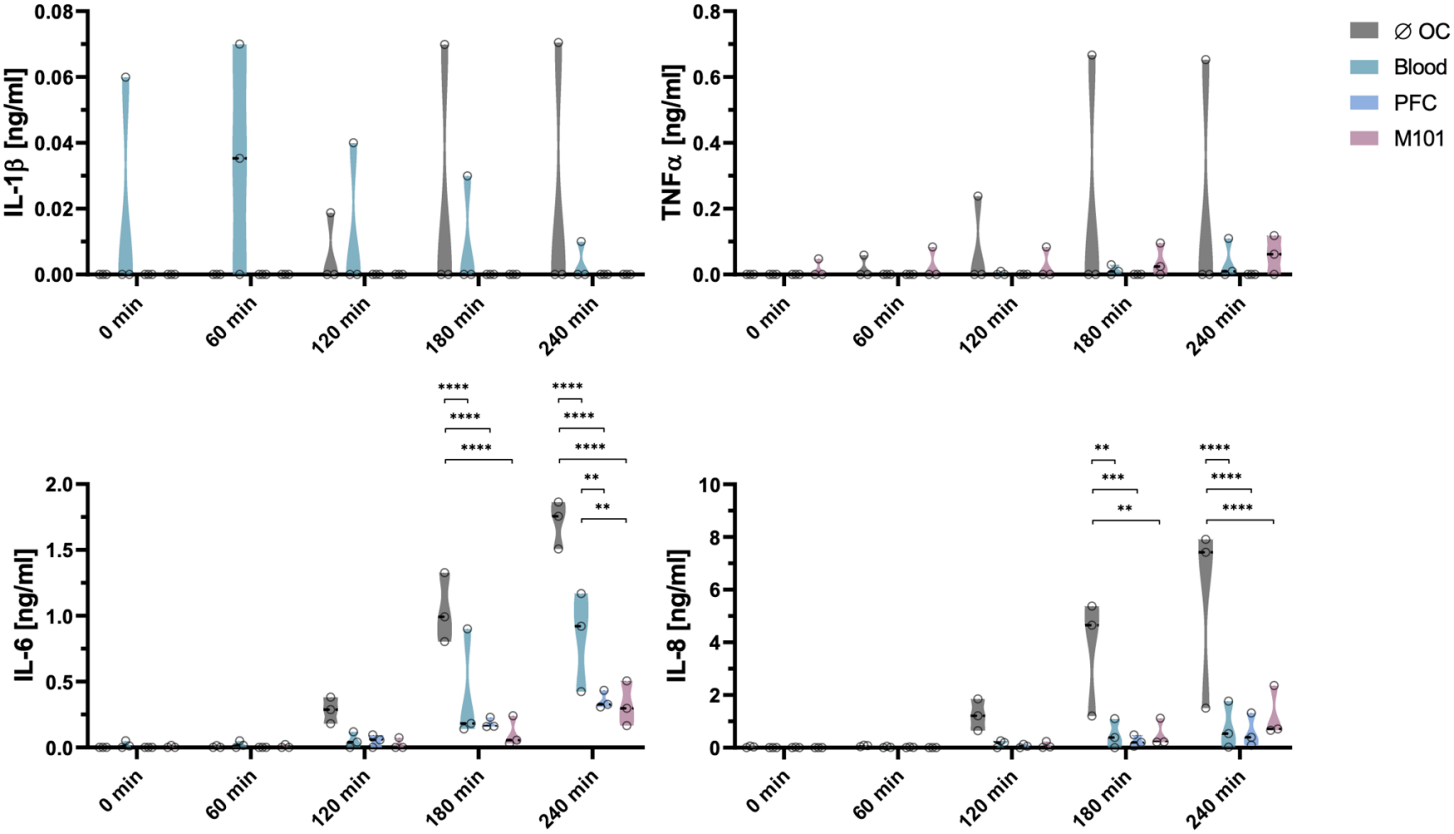
Cytokine secretion profile during normothermic EVKP using OCs. Concentrations of interleukin (IL)-1β, tumor necrosis factor (TNF)α, IL-6, and IL-8 were measured in perfusates of control kidneys and kidneys exposed to OCs at different time points (0, 60, 120, 180, and 240 min). Graphs represent means and standard deviations (*n *= 3). IL-6 and IL-8 levels decreased significantly after addition of OCs (**p < 0.01; ***p < 0.001; ****p < 0.0001; two-way ANOVA).

Cytokines are important immunomodulators playing a crucial role after transplantation by regulating immune responses and differentiation of immune cell populations (40). To evaluate the inflammatory status of the kidney after EVKP, levels of IL-1β, TNFα, IL-6, and IL-8 secretion were analyzed. During EVKP, IL-1β and TNFα levels remained low in all groups over time. Release of IL-6 and IL-8 was upregulated in the group of kidneys perfused without OCs and showed the highest values after four hours of EVKP. Remarkably, the addition of OCs contributed to a significant reduction of cytokine levels after three hours of EVKP when compared to the control kidneys perfused without OCs. In contrast to the control group without OCs (1.04 ± 0.27 ng/ml), only 0.18 ± 0.04 ng/ml (*p *< 0.0001) and 0.11 ± 0.11 ng/ml (*p *< 0.0001) IL-6 was detected in the perfusion solutions of the PFC-based OC and M101 group, respectively. Similarly, IL-8 release was significantly decreased to 0.25 ± 0.22 ng/ml (*p *< 0.01) with PFC-based OCs and 0.52 ± 0.52 ng/ml (*p *< 0.001) with M101, while 3.75 ± 2.23 ng/ml IL-8 was measured in the perfusate of the control group without OCs. In the group of kidneys perfused with blood, IL-6 (0.41 ± 0.43 ng/ml; *p *< 0.0001) and IL-8 (0.50 ± 0.56 ng/ml; *p *< 0.01) levels were also significantly reduced compared to the control group without OCs (Figure 4). These results indicate that supplementation of organ perfusion solutions with OCs contribute to decrease the pro-inflammatory environment of the kidney by preventing the release of pro-inflammatory cytokines.

### 3.5. Effect of OCs on the expression of inflammatory markers

**Figure 5:**
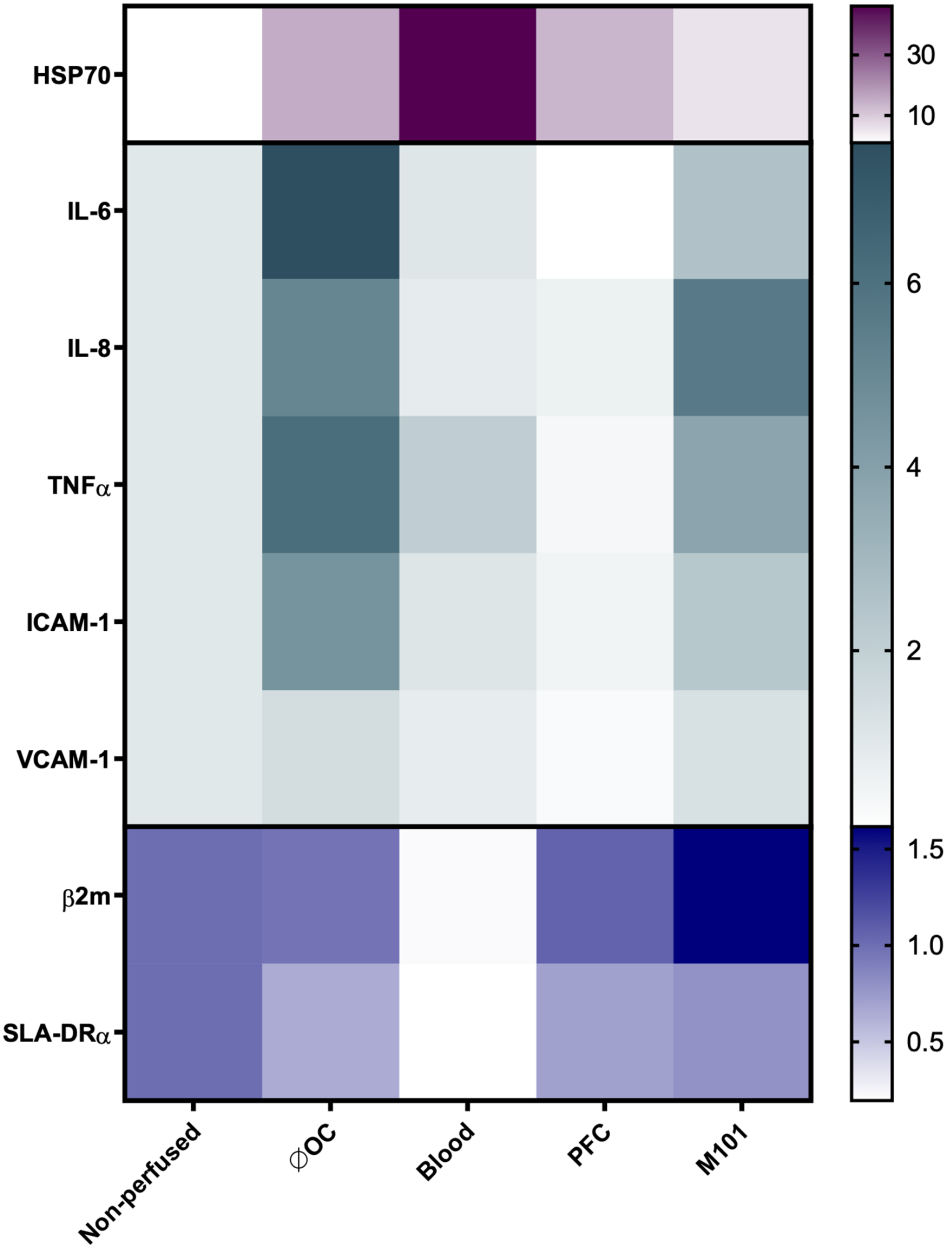
Heatmap representing the gene expression profile of *ex-vivo* perfused kidneys using OCs. Relative quantification (RQ) of heat shock protein 70 (HSP70), IL-6, IL-8, TNFα, intercellular adhesion molecule 1 (ICAM-1), vascular cell adhesion molecule 1 (VCAM-1), β2-microglobulin (β2 m), and swine leukocyte antigen-DRα (SLA-DRα) gene expression in renal tissues measured by qPCR. The data were normalized to the endogenous housekeeper gene GAPDH. Color intensity represents the up- and downregulation of mRNA expression values relative to the control tissue of non-perfused kidneys. Compared to the kidneys perfused without OCs, IL-6 and ICAM-1 gene expression levels significantly decreased after addition of OCs (*p < 0.05; **p < 0.01; ***p < 0.001; ****p < 0.0001; one-way ANOVA).

Tissue immunogenicity is defined by the antigen presentation capacity determined by the level of major histocompatibility complex (MHC) expression, cytokines, and chemokines involved in the leukocyte recruitment and activation as well as by adhesion molecules that support their adhesion and extravasation into the graft. Hence, upregulation of such genes may contribute to a poor graft outcome by supporting allogeneic immune responses and graft rejection (41). In comparison to the control kidneys perfused without OCs (7.54 ± 0.43), the addition of OCs contributed to a significantly reduced IL-6 expression in the renal tissue indicated by RQ values of 1.08 ± 0.60 (*p *< 0.0001) for blood, 0.08 ± 0.03 (*p *< 0.0001) for PFC-based OCs, and 2.58 ± 0.48 (*p *< 0.0001) for M101 perfused kidneys (Figure 5). These results confirmed the previous observations, showing decreased IL-6 levels in the perfusate during EVKP. Interestingly, also ICAM-1 transcript levels were significantly downregulated in kidneys perfused with blood (1.13 ± 1.34; *p *< 0.01), PFC-based OCs (0.52 ± 0.09; *p *< 0.001), and M101 (2.35 ± 0.22; *p *< 0.05) when compared to control kidneys perfused without OCs (4.54 ± 0.99). For IL-8, TNFα, and VCAM-1 expression, similar tendencies were observed in the renal tissues. SLA class I levels were assessed by quantification of β2 m transcripts. SLA class I transcript levels were upregulated in the tissue of kidneys perfused with PFC-based OCs (1.06 ± 0.07) and M101 (1.61 ± 0.46) in comparison to the levels detected in the tissue of kidneys perfused without OCs (0.96 ± 0.84). In contrast, kidneys perfused with blood showed decreased SLA class I gene expression levels (0.22 ± 0.15). Similarly, SLA class II (SLA-DRα) transcript levels were slightly higher in kidneys perfused with PFC-based OCs (0.70 ± 0.14) and M101 (0.80 ± 0.12) compared to those detected in kidneys perfused without OCs (0.65 ± 0.28). Kidneys perfused with blood demonstrated decreased SLA class II gene expression levels (0.20 ± 0.12; *p *< 0.05). HSP70 transcripts were upregulated in all groups, with the highest increase detected after EVKP with blood (46.00 ± 27.16), while control kidneys perfused without OCs (15.12 ± 21.86) as well as PFC-based OCs (13.58 ± 10.32) and M101 (5.63 ± 1.05) perfused kidneys exhibited lower HSP70 gene expression levels (Figure 5).

## 4. Discussion

Normothermic *ex-vivo* organ perfusion offers the opportunity to uphold the graft in a near to physiological state and reduce the cold ischemic time (5, 6). In the literature, normothermic preservation strategies differ significantly, ranging from one hour to 24 h EVKP. Preserving a donor organ under normothermic conditions has many advantages, including the restoration of an aerobic metabolism, the repair of reversible injuries, the possibility to assess organ quality, and the opportunity to introduce specific pharmacological or genetic engineering therapies to a functioning organ to improve transplantation outcome (42, 43). It is hypothesized that machine perfusion upregulates protective repair mechanisms that may mitigate ischemia-reperfusion injury. HSP70 expression was found to be increased under perfusion conditions, which contributes to tolerance by reducing stress-induced denaturation and aggregation of intracellular proteins (42). Our results demonstrate a reduced increase in HSP70 expression during EVKP in presence of PFC-based OCs and M101, which may suggest that these organs were exposed to less stress conditions. In contrast, kidneys perfused with blood revealed the highest HSP70 gene expression. Previous studies have shown a correlation between elevated HSP70 levels and the degree of hemolysis (44). RBC concentrates are known to be associated with several logistical and biophysical limitations, including a short shelf life, limited availability, and hemolysis during prolonged perfusions, especially in centrifugal and rotatory pump systems (30, 45). Free hemoglobin rapidly dissociates into monomeric and dimeric forms, which leads to nitric oxide (NO) scavenging after extravasation into the subendothelial domain of the vascular system. Reduced NO concentrations cause vasoconstriction and hypertension. Thus, NO scavenging may increase the VR and pressure of the perfusion system, resulting in decreased flow rates and hypoperfusion of the organ. Additionally, free hemoglobin is converted into methemoglobin, showing a much lower oxygen affinity (46, 47). To avoid these undesirable effects of free hemoglobin, alternative OCs have been widely evaluated.

Levels of lactate and AST are considered as markers for hypoxia and cell damage (2, 39). The increase in AST activity levels during perfusion indicates tissue injury, which has been reported previously in studies focusing on *ex-vivo* recirculating organ perfusions. It might be caused by cell damage during periods of warm ischemia prior perfusion or the use of different perfusion pumps (48, 49). EVKPs with OCs do not result in a significant AST increase, suggesting no additional tissue damage induced by OCs. This is in line with former data on rat EVKP with PFC-based OCs (10). Elevated lactate production indicates an insufficient supply of oxygen to renal cells preventing oxidative phosphorylation and resulting in an anaerobic metabolism (50, 51). Lactate levels were measured to assess the renal cell metabolism. Under normoxia, lactate concentrations of approximately 2.53 ± 1.10 mM are detected in Göttingen minipigs and may increase in response to external stress such as exsanguination (52, 53). During EVKP using blood, lactate levels did not increase suggesting for the maintenance of an aerobic metabolism and a sufficient oxygen supply. Remarkably, in comparison to EVKP in absence of OCs, PFC-based OCs and M101 were capable to significantly decrease lactate concentrations. Thus, both OCs promote oxygen supply to renal cells and facilitate aerobic cell metabolism. Hence, OCs might support long-term EVKPs using acellular perfusion solutions.

A major aim of this study was to evaluate the effect of the OCs on organ immunogenicity. Even if the perfusion conditions are supposed to be protective, the artificial setting can induce a local inflammatory response, which is associated with a poor clinical outcome after transplantation (29, 54, 55). MHC expression on the graft may trigger potent allogeneic immune responses contributing to rejection. Hence, levels of MHC class I and II expression in the graft will influence transplantation outcome. Previous studies indicated a reduced tissue expression of MHC class II by oxygenated machine perfusion as compared to SCS, minimizing functional alterations and immunogenicity of a graft (56). Our results corroborated those observations, but interestingly they show higher transcript levels of SLA class II with PFC-based OCs and M101 in comparison to those detected in kidneys perfused with blood. Further studies will be necessary to evaluate the impact on cellular and humoral responses after transplantation.

Studies on EVKP reported a major inflammatory storm with high concentrations of IL-6, IL-8, and IFN-γ after perfusion, leading to mobilization of donor leukocytes. The pro-inflammatory environment of the donor kidney might provide insights into the immune responses that occur immediately following transplantation (28, 29). Perfusion with both OCs reduced the pro-inflammatory cytokine storm during EVKP. Previously, a decrease of cytokine secretion in presence of OCs had been reported during *ex-vivo* lung perfusion (57, 58). The release of pro-inflammatory cytokines such as IL-1β, IL-6, IL-8, and TNFα may orchestrate endothelial permeability and neutrophil infiltration during reperfusion, driving a direct allorecognition and recipient immune activation following transplantation. In particular, IL-6 is a relevant marker for graft survival after KTx. IL-6/IL-6R activates signaling pathways involved in inflammation, ischemia-reperfusion injury, cellular and humoral immune responses, and fibrosis, causing allograft injury (55). Acute and chronic kidney allograft rejection promoted by IL-6 is well described. In a murine KTx model, renal expression of IL-6 was upregulated and associated with decreased intragraft Foxp3+ Tregs, leading to subsequent allograft rejection. On the other hand, lack of graft-produced IL-6 significantly prolonged renal allograft survival (59). Studies of human kidney transplant recipients have found increased levels of IL-6 in the serum, urine, and biopsy tissue linked to inflammatory cell infiltration during renal allograft rejection (60). Our results showed a reduction of IL-6 concentration in the perfusates of kidneys perfused with OCs, indicating for a lower immunogenic potential of the organ in comparison to those perfused without OCs. IL-8 is another important inflammatory mediator and chemotactic cytokine associated with prognostic potential to predict rejection after KTx. IL-8 is produced by damaged endothelium as well as macrophages, T-cells, and renal tubular epithelial cells following the spread of inflammation. A strong inflammatory response and high IL-8 levels are related to allograft dysfunction and loss (61). Kidneys perfused with blood or PFC-based OCs demonstrated lower levels of IL-8 in the perfusate in comparison to those perfused without OCs. Inflammatory responses are also mediated by the upregulation of endothelial adhesion molecules (ICAM-1 and VCAM-1), promoting leucocyte-endothelial interactions and subsequently microvascular congestion. Consequently, it may cause an increase in cytokine levels, oxygen radicals production, and activation of the complement system, which sustains the injury response (62). These results outline the importance of mitigating inflammation during *ex-vivo* organ perfusion. Altogether, OCs improved the perfusion solution by decreasing the secretion of pro-inflammatory mediators and expression of adhesion molecules on both transcriptional and protein level, reducing the immunogenic potential of the graft.

As already mentioned, several preclinical studies support the beneficial effects of alternative OCs in the field of *ex-vivo* organ perfusion (10, 19, 20, 25, 26, 56, 58). PFC-based OCs and M101 provide a high oxygen-carrying capacity and perform well in cell metabolism and histology with an amelioration of ischemia-reperfusion injury (21, 27). Especially, PFC-based OCs are characterized by an environmentally insensitive oxygen uptake and release and low production costs. However, a high oxygen partial pressure is needed to maximize oxygen content of PFC-based OCs, which is easily addressed during *ex-situ* machine perfusion. Furthermore, the intravascular use of PFCs requires the formulation as emulsion, which is realized in actually used PFC-based OC such as albumin-derived perfluorodecalin-filled artificial OCs or dodecafluoropentane emulsion with the physiological emulsifier albumin (22). These emulsions are compatible with aqueous perfusion solutions and supply sufficient oxygen to protect organs during EVKP (10) and in whole animal hemodilution scenarios (95% blood draw with subsequent exchange to PFC-based OCs in a rat massive hemodilution study) (63). As mentioned above, oxygen loading and unloading occur twice as fast as in RBCs and the oxygen extraction rate is 3-fold higher. While RBCs bind more oxygen than a PFC-based OC at the same oxygen partial pressure, they release less of it. The oxygen extraction rate of RBCs at physiologically relevant gradients (e.g., from 80 mmHg in the lung to 40 mmHg in the tissue) reaches 25% and significantly decreases during storage due to a loss of 2.3 bisphosphoglycerate. In contrast, oxygen release from PFC-based OCs remains constant during storage and reaches 90% under the same conditions, since the van der Waals oxygen-PFC interaction is one magnitude lower than the covalent oxygen-Fe^2+^ interaction present in hemoglobin (64). These different binding properties also lead to a faster oxygen release from perfluorocarbons than from hemoglobin (22, 64). M101 can carry up to 156 molecules of oxygen when saturated, resulting in a 39 times high oxygen-binding capacity than mammal hemoglobin. Furthermore, M101 is derived from a poikilotherm invertebrate (*Arenicola marina* does not control its internal temperature), which allows its hemoglobin to function in hypothermia and normothermia, unlike RBCs. Accordingly, M101 releases oxygen over a broad range of temperatures according to a gradient and demonstrates functionality without being packaged into RBCs or other membranes. So far, M101 has only shown preliminary evidence in preclinical hypothermic machine perfusion trials and clinical SCS studies but has not yet been used during normothermic *ex-vivo* organ perfusions (32). Nonetheless, by reducing the production of pro-inflammatory mediators, alternative OCs may offer a suitable replacement to hemoglobin in RBCs as natural OC.

In conclusion, our findings indicate that blood, PFC-based OCs, and M101 decrease the pro-inflammatory status of the organ acquired during EVKP. Hence, both PFC-based OCs and M101 are promising alternative OCs to RBC, supporting normothermic EVKP and simultaneously contributing to reduce the immunogenic status of the graft.

## Data Availability

The raw data supporting the conclusions of this article will be made available by the authors upon reasonable request.
