# Supplementary material for: Oxygen carriers affect kidney immunogenicity during ex-vivo machine perfusion
Source: Front Transplant. 2023 Jun 16;2:1183908. doi: 10.3389/frtra.2023.1183908 (PMC11235266; doi:10.3389/frtra.2023.1183908)
Supplement: Supplementary file 1 [file Image1.pdf]

Supplementary Material

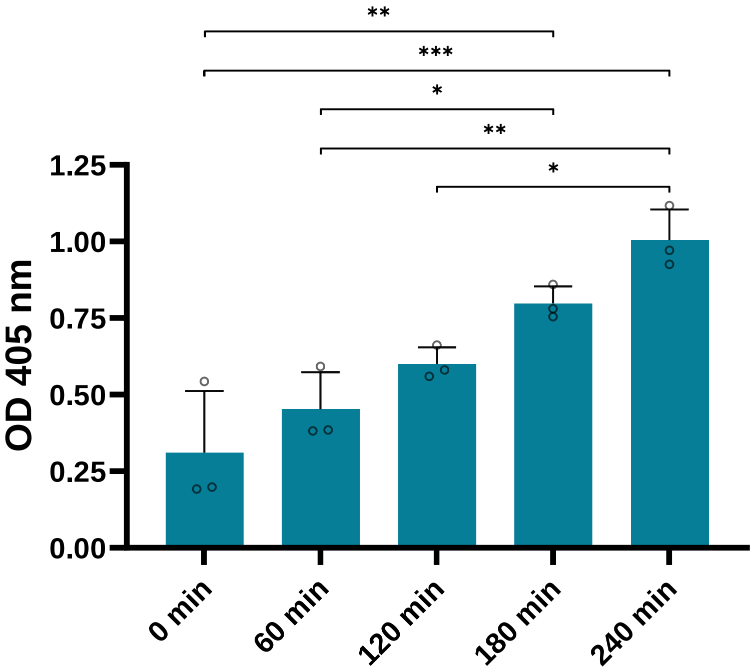

Supplementary Figure 1. Hemolysis levels during normothermic EVKP with red blood cells (RBCs) as OC. Hemolysis level of RBCs was quantified in perfusates of kidneys exposed to blood (group 2) at different time points (0, 60, 120, 180, and 240 min) by detecting free hemoglobin. Absorbance at 405 nm was measured with the Synergy 2 Multi-detection microplate reader and optical density (OD) units were corrected by background subtraction (STEEN/Ringer-based solution). Graphs represent means and standard deviations (n=3). Hemolysis level significantly increased over time during EVKP (*$p<0.05$; **$p<0.01$; *******$p<0.001$; one-way ANOVA).
